# Prophylactic Cefazolin Dosing in Obesity—a Systematic Review

**DOI:** 10.1007/s11695-022-06196-5

**Published:** 2022-07-09

**Authors:** Matthew Coates, Alison Shield, Gregory M. Peterson, Zahid Hussain

**Affiliations:** 1 Faculty of Health, University of Canberra, 1 Kirinari Street, Bruce, Canberra, ACT 2617 Australia; 2 School of Pharmacy and Pharmacology, University of Tasmania, Hobart, Australia

**Keywords:** Obesity, Cefazolin, Dosing, Surgical site infection, Prophylaxis

## Abstract

Currently, there is no consensus on whether a standard 2-g prophylactic cefazolin dose provides sufficient antimicrobial coverage in obese surgical patients. This systematic review analysed both outcome and pharmacokinetic studies, aiming to determine the appropriate cefazolin dose. A systematic search was conducted using 4 databases. In total, 3 outcome and 15 pharmacokinetic studies met the inclusion criteria. All 3 outcome studies concluded that there is no need for increased dose. Also, 9 pharmacokinetic studies reached this conclusion; however, 6 pharmacokinetic studies recommended that 2-g dose is insufficient to achieve adequate plasma or tissue concentrations. The stronger body of evidence supports that 2-g dose of cefazolin is sufficient for surgery lasting up to 4 h; however, large-scale outcome studies are needed to confirm this evidence.

**Supplementary Information:**

The online version contains supplementary material available at 10.1007/s11695-022-06196-5.

## Introduction

Globally, more than 600 million people are living with obesity, and this number is continuing to rise [1]. Obesity increases the likelihood of developing diabetes, coronary heart disease and many other chronic illnesses [2], and individuals with obesity are more likely to require surgical procedures at earlier stages of life. Furthermore, patients with obesity who undergo surgery are at greater risk of developing intra- and post-surgical complications, such as venous thromboembolism [3], delayed wound healing [4] and surgical site infection (SSI) [5, 6]. SSI can be of particular concern, as it reduces survival rates, increases disease-related morbidity, increases duration of hospital stay and increases the likelihood of hospital re-admissions. Combined, these factors result in significant costs to the healthcare system [7]. To minimise the risk of SSI, it is standard procedure to administer intravenous prophylactic antibiotics prior to surgery from clean-contaminated surgery and beyond.

Patients with obesity have significant physiological changes such as altered renal function and body volume and composition, which in turn can alter the pharmacokinetic parameters of many drugs [8]. This can potentially reduce the efficacy of a standard dose for non-obese individuals [9]. As such, dose adjustments are often warranted in this patient population. Cefazolin, a hydrophilic beta-lactam antibiotic, is widely used in SSI prophylaxis [10–12]. A standard dose of cefazolin is 2 g given as an intravenous bolus within 15–60 min prior to surgery. Studies have aimed to determine whether this dose is efficacious in patients with obesity, or whether practice guidelines should be updated to include a higher recommended dose for this group of patients. These studies fall into two broad categories: clinical outcome studies and pharmacokinetic studies. Outcome studies assess the incidence of SSI, usually 30 days following surgery, aiming to ascertain whether a standard 2-g dose of cefazolin was sufficient in preventing SSI in patients with obesity. Pharmacokinetic studies measure the cefazolin concentrations in plasma and/or body tissues before and during surgery. These drug tissue concentrations are compared with a pre-determined target minimum inhibitory concentration (MIC) to assess adequacy of dosing.

Although many clinical and experimental studies have been conducted to examine whether patients with obesity need higher cefazolin dosing, no systematic review has combined both outcome and pharmacokinetic studies to draw a rigorous conclusion. Therefore, the aim of this systematic review was to gather and critically evaluate the current data surrounding prophylactic cefazolin dosing in patients with obesity, informing evidence-based clinical practice.

## Methods

### Information Sources and Search Strategy

CINAHL, Medline, PubMed and Scopus electronic journal databases, since date of inception, were accessed and searched during April 2021. The search included the following key words: cefazolin, obese (or obesity or overweight), surgery (or surgical or elective or procedure) and prophylaxis (or prophylactic or dose). This review followed Preferred Reporting Items for Systematic Reviews and Meta-Analyses (PRISMA) guidelines [13] (Appendix 1) and was registered with PROSPERO (CRD42021276409).

### Article Selection and Review

After removal of duplicates, article titles and abstracts were screened by one reviewer (Author 1). Following this, a full-text review was conducted by Author 1 and checked by Author 4. Author 2 was consulted in case of conflicts.

### Inclusion Criteria

Human studies in which patients underwent elective surgical procedure and received cefazolin as the sole prophylactic antibiotic agent were included. Studies were also required to have at least one group of patients with obesity (BMI ≥ 30 kg/m^2^). Studies which investigated the adequacy of the prophylactic cefazolin dose in terms of either rate of surgical site infection (SSI) or cefazolin plasma/tissue concentrations were retained. These studies reported BMI and cefazolin dose and one or more of the following: rates of SSI post-surgery, cefazolin tissue/plasma concentrations, MIC, protective duration, elimination half-life (t_1/2_) and area under the curve (AUC). Studies in which patients received antibiotics other than cefazolin, or studies in which patients received additional antibiotics for treatment of infection (not for prophylaxis), were excluded.

### Quality Assessment

To evaluate quality of evidence, articles which met the inclusion criteria were assessed using the JBI critical appraisal tools [14]. This process was adopted to determine the possibility of biases or inadequacies in study design, data collection and analysis by assigning a score to each article based on a multi-item checklist. The quality assessment was conducted by two independent reviewers (MC and ZH). Disagreements between these two reviewers were to be settled by a third reviewer (AS). Studies that satisfied the criteria for greater than 60% of the checklist items were included in the systematic review (Appendix 2–4).

### Data Extraction

Selected articles were grouped into two categories: outcome study or pharmacokinetic study. The following data was extracted from the articles: authors, article title, publication year, study design, type of surgery, study population, number of participants (*N*), intervention and comparator, diagnostic criteria, study outcomes and conclusions made by the authors. This information is tabulated (Appendix 5–6).

## Results

### Study Selection

**Fig. 1:**
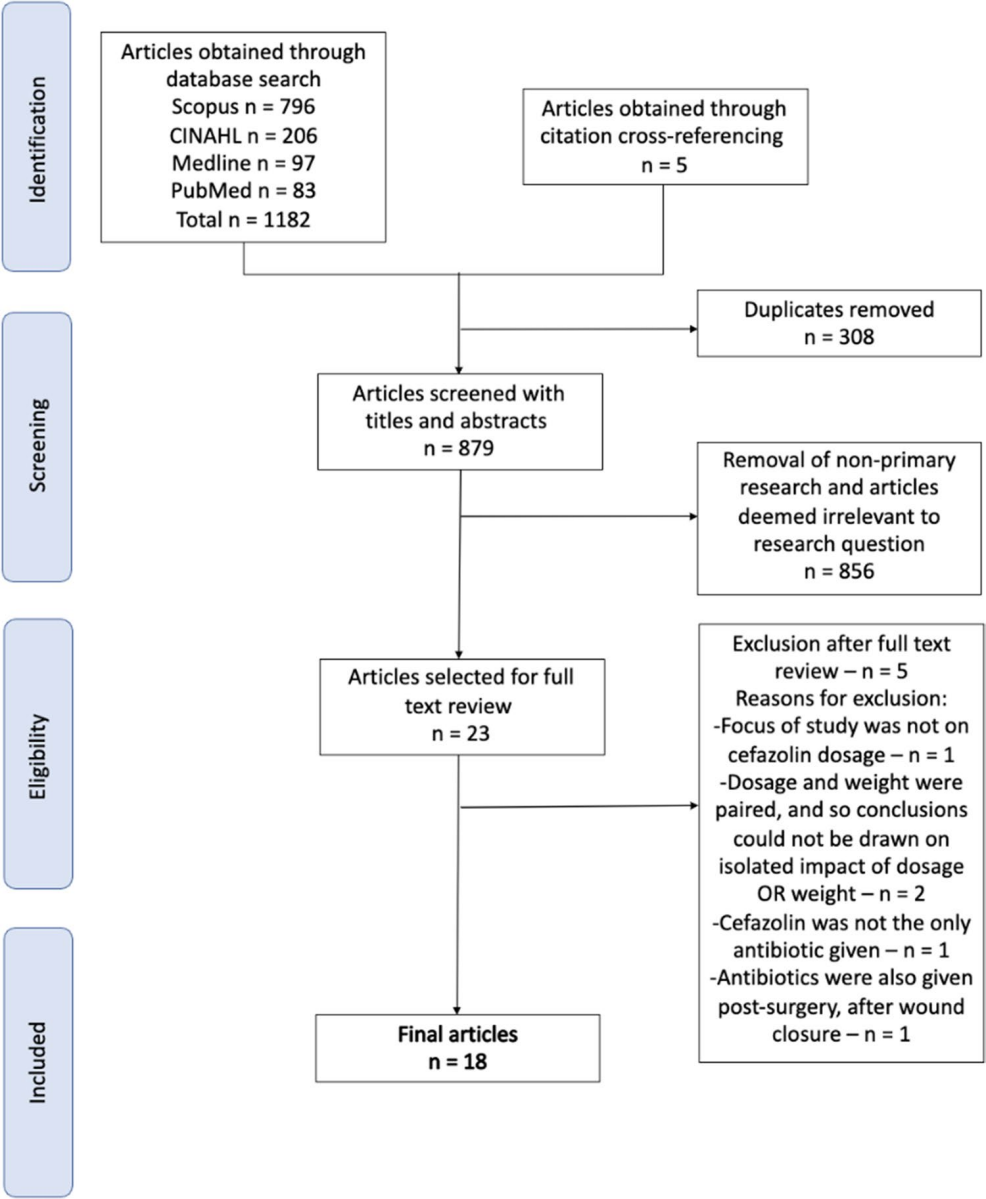
PRISMA diagram outlining study selection process

The PRISMA flow diagram is presented in Fig. 1. In total, 1182 results were identified, of which, 13 articles met the inclusion criteria and five additional articles were identified from cross-reference search. In total, 18 articles were included in this systematic review and all 18 met the pre-defined cutoff criteria (> 60% of checklist items) for quality assessment. The major reasons for exclusion of articles after full text were the following: antibiotics other than cefazolin were given, additional doses of cefazolin were administered post-surgery or cefazolin dosage given was based on patient weight, meaning that impacts of dose or patient weight could not be isolated from one another.

**Fig. 2:**
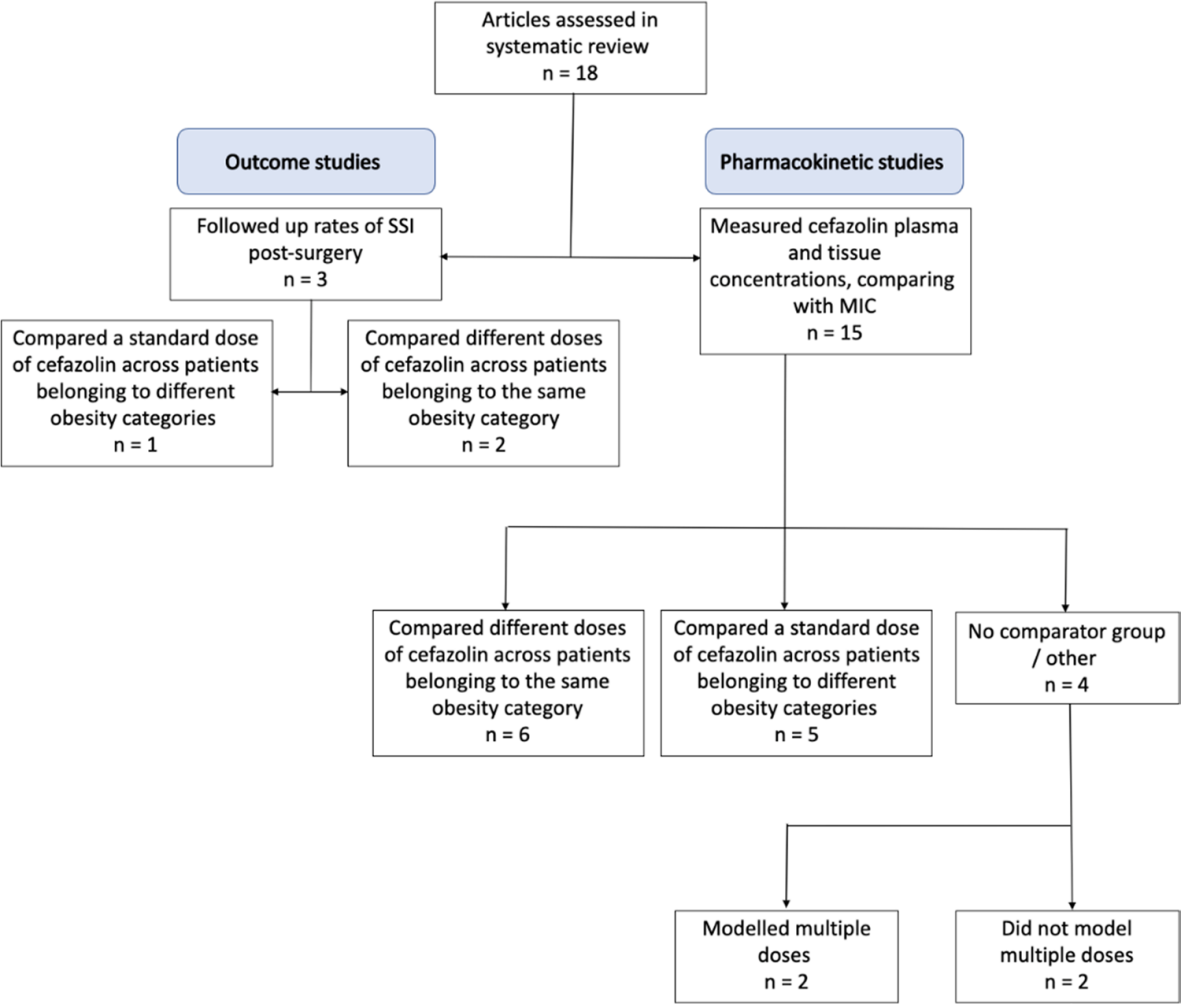
Study designs

Selected articles were categorised into outcome studies or pharmacokinetic studies (Fig. 2). Outcome studies were further subdivided into (a) dosing comparator (2 g vs 3 g) and (b) non-obese comparator (obese vs non-obese). Pharmacokinetic studies were sub-divided into (a) dosing comparator (2 g, 3 g or 4 g), (b) obese comparator (obese vs morbidly obese vs super morbidly obese) and (c) studies that only had one group and drew conclusions based on the pre-defined pharmacokinetic parameter ranges, or through techniques such as Monte Carlo simulations [15]. Two studies [16, 17] combined approaches (a) and (b).

### Outcome Studies

#### Dosing Comparator Studies

All outcome studies used the Centre of Disease Control and Prevention definition of SSI. Two of the three outcome studies investigated the efficacy of doses above 2 g cefazolin for SSI prevention [18, 19]. Both retrospective studies assessed large cohorts (*N* = 335 and *N* = 436, respectively) and compared cefazolin 2 g with 3 g (Table 1). The first study [18] found the SSI occurrence in post-caesarian patients with obesity was 13.1% in both groups, regardless of dose. The second [19] found SSI rates of 7.2% with 2 g and 7.4% with 3 g dosing (*p* = 0.95) in patients who underwent various elective procedures. Perhaps notably, there was a mean BMI difference of 3–4 kg/m^2^ between control and intervention groups in both studies (Table 1).

**Table 1:** Key findings of outcome studies

Dosing comparator						
Study	Surgery type	Participants (***N***)	Control	Intervention	Outcome	2-g dose sufficient?
Ahmadzia et al. 2015 [18]	Caesarean section	335	2 g of cefazolin, mean BMI 49.9 kg/m^2^	3 g cefazolin, mean BMI 53.0 kg/m^2^	No significant difference in SSI rates (*p* = 0.996)	Yes
Peppard et al. 2016 [19]	Elective surgery (multiple)	436 (152 and 284)	2 g of cefazolin, mean BMI 36.4 kg/m^2^	3 g of cefazolin, mean BMI 40.1 kg/m^2^	SSI rates of 7.2% in 2 g and 7.4% in 3 g dosing groups. No significant difference was found (*OR* = 0.98, *p* = 0.95)	Yes
**Weight-based comparator**						
Hussain et al. 2019 [20]	Elective surgery (multiple)	304 (152 and 152)	2 g of cefazolin, patients without obesity (BMI < 30 kg/m^2^)	2 g of cefazolin, patients with obesity (BMI > 30 kg/m^2^)	Trend towards increased SSI in obese group, but not significant (*p* = 0.25)	Yes

summarises key information relating to outcome studies including doses, BMI information and conclusions drawn

#### Weight-Based Comparator Studies

The only weight-based comparator study included in this review evaluated the efficacy of 2-g cefazolin in cohorts with (*N* = 152) and without obesity (*N* = 152) (Table 1) [20]. This study found higher SSI rates in the cohort with obesity compared to the non-obese cohort, although this difference was not statistically significant (8.6% vs 4.6%; *p* = 0.25).

### Pharmacokinetic Studies

All pharmacokinetic studies compared measured cefazolin concentration with a pre-determined MIC target value. As MIC values for a given antimicrobial depend on which microbes it is being tested against, there was some variability between studies for MIC targets [21]. Timing of tissue sampling and type of tissue used for sampling (for example, samples taken from interstitial fluid versus those taken from adipose tissue) also varied between studies, potentially affecting drug tissue concentrations and expected targets [22].

#### Dosing Comparator Studies

Six pharmacokinetic studies directly investigated cefazolin doses above 2 g: five used 3 g, [16, 17, 23–25] whilst one used 4 g. [26] Four studies measured cefazolin concentrations in adipose tissue [17, 23, 25, 26], whilst two looked at plasma and interstitial tissue [16, 24]. The results of these studies are summarised in Table 2.

**Table 2:** Key findings of pharmacokinetic studies—dosing-based comparator

Study	Surgery type	Participants (*N*)	Control	Intervention	MIC	Measured tissue	Outcome	2-g dose sufficient?
Ho et al. 2012 [16]	Elective surgery	25 (10, 5, 5, and 5)	2 g cefazolin IV push, mean BMI 44.1 kg/m	2 g cefazolin 30 min infusion, mean BMI 43.7 kg/m^2^; Group III: 2 g IV infusion, mean BMI 55.7 kg/m^2^; Group IV: 3 g infusion, mean BMI 55.2 kg/m^2^	8 mcg/mL	Interstitial/plasma	The mean cefazolin concentrations after 30 min were similar in all groups. Half-life was unaffected by administration methods	Yes
Stitely et al. 2013 [26]	Caesarean section	20 (11 and 9)	2 g of cefazolin, mean BMI 46.0 kg/m^2 a^	4 g of cefazolin, mean BMI 43.2 kg/m^2 a^	4 mcg/g	Adipose	Tissue concentrations were significantly higher in the 4-g dosage group (*p* = 0.0005), but all patients in both groups remained above MIC	Yes
Young et al. 2015 [23]	Caesarean section	26 (13 and 13)	2 g of cefazolin, mean BMI 42.9 kg/m^2 a^	3 g of cefazolin, mean BMI 41.8 kg/m^2 a^	1 mcg/g, 4 mcg/g	Adipose	The 3-g group had higher cefazolin concentrations (*p* = 0.02), but concentrations were above MIC in all patients in both groups	Yes
Maggio et al. 2015 [24]	Caesarean section	57 (28 and 29)	2 g of cefazolin, mean BMI 38.9 kg/m^2 a^	3 g of cefazolin, mean BMI 39.3 kg/m^2 a^	2 mcg/g, 8 mcg/g	Adipose	Patients given 2 g dose were above MIC of 8 mcg/g 61% of the time, whilst for 3 g it was 72% (*p* = 0.12). All patients were above MIC of 2 mcg/g	Yes
Swank et al. 2015 [17]	Caesarean section	57 (28 and 29)	2 g of cefazolin^b^	3 g of cefazolin^c^	8 mcg/mL	Adipose	With 2 g of cefazolin, only 20% of the cohort with a BMI of 30–40 kg/m^2^ and none of the cohort with a BMI of > 40 kg/m^2^ reached MIC	No
Palma et al. 2018 [25]	Bariatric surgery (gastric bypass or sleeve gastrectomy)	9 (4 and 5)	2 g of cefazolin, mean BMI 49.7 kg/m^2^	3 g of cefazolin, mean BMI 44.0 kg/m^2^	1 mcg/mL, 2 mcg/mL	Interstitial/plasma	2 g dose is sufficient for bacteria < 1 mg/L. For species requiring > 2 mg/L, 2 g is sufficient for up to 4 h, whilst 3 g gives better coverage after this	Yes

summarises key information relating to pharmacokinetic studies that tested different doses between groups, including doses, MIC targets named, tissue type that was sampled and conclusions drawn. ^a^At time of caesarian delivery. ^b^Historic cohort from Pevzner et al., [27]further subdivided into BMI < 30 kg/m2, BMI 30–40 kg/m2 and BMI > 40 kg/m2..^c^Further subdivided into 30–40 kg/m2 and > 40 kg/m2 groups

One of the studies that sampled from adipose tissue [23] found that, although the 3-g dosing group consistently had significantly higher adipose cefazolin concentrations compared to the 2-g dosing group (*p* = 0.02), both doses were sufficient in meeting 4-mcg/g MIC targets in all patients (cefazolin concentrations at closure were 10.0–14.0 mcg/g in the 2-g group and 13.0–23.0 mcg/g in the 3-g group). A second study [24] found that all patients who received either 2 g or 3 g gave tissue sample concentrations above MIC 2 mcg/g, although a higher 8 mcg/g MIC target was reached in only 61% and 72% of patients in the respective groups (4.4–13.2 mcg/g vs 6.7–18.8 mcg/g; *p* = 0.12). Still, the authors concluded that 2-g dosing was sufficient because the difference was not statistically significant. Another study compared 2-g and 4-g doses [26] and again found that both doses were sufficient in meeting the MIC target of 4 mcg/g in adipose tissue in all patients, despite higher mean tissue concentrations in the 4 g compared to the 2-g dosing group (34.9 mcg/g vs 21.7 mcg/g; *p* = 0.0005).

A study comparing 2 g of cefazolin given via IV push with (a) 2 g of cefazolin given via a 30-min infusion and (b) 3 g of cefazolin given via a 30-min infusion [16] found that each of these doses resulted in plasma concentrations above the pre-specified MIC of 8 mcg/mL for more than 4 h. Mean cefazolin plasma concentrations ranged from 22.9 mcg/mL in the 2-g IV push group with BMI 40–50 kg/m^2^ to 40.8 mcg/mL in the 3-g infusion group in patients with BMI > 50 kg/m^2^. They also concluded that the method of administration did not affect the cefazolin elimination half-life. Another study which combined experimental and computational methods [25] measured the difference between 2-g and 3-g doses in plasma and subcutaneous tissue concentrations and used this data to run Monte Carlo simulations [15]. This study showed that 2-g cefazolin dose provided plasma and tissue concentrations above the pre-specified MIC of 2 mcg/mL for up to 4 h in 89% of patients, whilst 3 g kept concentrations above MIC for up to 5 h. It was concluded that a 2-g dose was sufficient for procedures lasting up to 4 h.

Conversely, a study [17] comparing a 3-g cefazolin dose with a historic cohort which received 2 g [27] found that the higher dose provided a greater chance of reaching MIC targets. This study reported that only 20% of the cohort with a BMI of 30–40 kg/m^2^ and none of the patients with a BMI of > 40 kg/m^2^ reached a plasma MIC of 8 mcg/mL with 2-g cefazolin dosing. All patients in the BMI 30–40 kg/m^2^ group and 71% in the > 40 kg/m^2^ group reached target concentrations with a 3-g cefazolin dose.

#### Weight-Based Comparator Studies

The five pharmacokinetic studies which used weight-based comparators are summarised in Table 3. Out of these studies using the standard 2-g cefazolin, one measured cefazolin concentration in adipose tissue [27]. This study found that samples from 20% of patients with BMI 30–40 kg/m^2^ and 44% of patients with BMI > 40 kg/m^2^ did not meet a MIC of 4 mcg/g at closure. Significant differences in mean cefazolin concentrations between BMI categories were found at time of incision (*p* = 0.009 and *p* < 0.001), but not at time of closure (*p* = 0.36 and *p* = 0.07).

**Table 3:** Key findings of pharmacokinetic studies—weight-based comparator

Study	Surgery type	Participants (*N*)	Control	Intervention		MIC	Measured tissue	Outcome	2-g dose sufficient?
Edmiston et al. 2004 [31]	Bariatric surgery (gastric bypass)	38 (17, 11 and 10)	BMI 40–49 kg/m^2^	BMI 50–59 kg/m^2^	BMI > 60 kg/m^2^	32 mcg/mL	Interstitial/plasma	Serum antimicrobial concentrations exceeded resistance breakpoint in 73%, 68% and 52% of BMI groups 40–49 kg/m^2^, 50–59 kg/m^2^ and > 60 kg/m^2^, respectively	No
Pevzner et al. 2011 [27]	Caesarean section	29 (10, 10 and 9)	BMI < 30 kg/m^2^	BMI 30–40 kg/m^2^	BMI > 40 kg/m^2^	1 mcg/g, 4 mcg/g	Adipose	20% and 44% of patients in obese and very obese categories, respectively, were not above a MIC of 4 mcg/g at wound closure At incision: -BMI < 30 kg/m^2^ vs 30–40 kg/m^2^: *p* = 0.009 -BMI < 30 kg/m^2^ vs > 40 kg/m^2^: *p* < 0.001 At closure: -BMI < 30 kg/m^2^ vs 30–40 kg/m^2^: *p* = 0.36 -BMI < 30 kg/m^2^ vs > 40 kg/m^2^: *p* = 0.07	No
Anlicoara et al. 2014 [29]	Bariatric surgery (sleeve gastrectomy and gastric bypass)	18	BMI < 40 kg/m^2^ 2 g bolus + 1 g infusion cefazolin	BMI > 40 kg/m^2^ 2 g bolus + 1 g infusion cefazolin		4 mcg/mL	Interstitial/plasma	Though patients in the lower weight category had higher cefazolin concentrations, all patients in both groups remained above MIC	Yes
Brill et al. 2014 [30]	Bariatric surgery (gastric bypass and Toupet fundoplication)	15 (8 and 7)	BMI 20–30 kg/m^2^	BMI > 40 kg/m^2^		4 mcg/mL	Interstitial/plasma	Monte Carlo simulations showed that probability of attaining MIC was significantly lower in patients with obesity	No
Groff et al. 2017 [28]	Caesarean section	8 (4 and 4)	BMI < 25 kg/m^2^	BMI > 30 kg/m^2^		1 mcg/mL, 2 mcg/mL, 17 mcg/mL	Interstitial/plasma	Both groups remained above MIC	Yes

summarises key information relating to pharmacokinetic studies that grouped patients by weight category, including doses, MIC targets named, tissue type that was sampled and conclusions drawn

Four studies measured interstitial fluid and plasma concentrations [28–31]. The first of these [28] found that all patients given 2 g cefazolin before surgery attained a maternal plasma MIC of 17 mcg/mL for the duration of the procedure. A second study compared the plasma concentrations in patients who had BMI < 40 kg/m^2^ and BMI > 40 kg/m^2^ after administration of 3 g (2 g bolus plus 1 g slow infusion). Plasma cefazolin concentrations were 5.3–13.8 mcg/mL in the BMI < 40 kg/m^2^ group and 4.7–8.64 mcg/mL in the BMI > 40 kg/m^2^ group (*p* = 0.006), all above a MIC of 4 mcg/mL. [29]

A study which used a combination of experimental methods and Monte Carlo simulations to compare patients with BMI < 30 kg/m^2^ to patients with BMI > 40 kg/m^2^2, 30 showed that the probability of maintaining an interstitial fluid MIC of 4 mcg/mL after 4 h with a 2-g cefazolin dose was 66.3% in the morbidly obese group compared with 94.9% in the non-obese group. For a MIC of 2 mcg/mL, this probability was 95.6% and 99.7% in the respective groups. The authors concluded that higher doses are needed in patients with morbid obesity. Another study that only assessed patients with BMI > 40 kg/m^2^ found that patients who were given 2 g cefazolin prior to surgery and re-dosed during surgery had 73% chance of exceeding a MIC of 32 mcg/mL in the BMI 40–49 kg/m^2^ group, with this probability dropping to 52% in the BMI > 60 kg/m^2^ group. [31]

#### Studies of a Single Cohort with Obesity

Four studies which were conducted on a single group each are summarised in Table 4. One of these studies found that all 37 patients in their cohort (≥ 35 kg/m^2^) had adipose tissue concentrations above a MIC of 1 mcg/g, with a mean adipose tissue concentration of 8.8 mcg/g (*SD* = 5.1) [32]. A study with a sample of 20 patients, with BMI 38–79 kg/m^2^, showed that serum levels of cefazolin remained above a MIC of 1 mcg/mL for up to 4 h after dosing in all patients, and that rate of cefazolin clearance was not correlated with weight (*p* = 0.42) [33]. The other two studies used modelling techniques to predict the impact of different doses in a single patient group. The first study [34] found that two of their twelve participants did not reach target MIC plasma concentrations of 2 mcg/mL when given 2 g of cefazolin. This study predicted (using simulations) that > 95% of patients who weigh 90–150 kg would have concentrations above this MIC if 2 g cefazolin was re-dosed at 2 h. Furthermore, > 99% of patients would reach targets if patients were instead treated with 3-g doses initially and again at 2 h. A final study administered 4-g cefazolin dose to patients with BMI > 40 kg/m^2^. It was found that this dose was sufficient to maintain a MIC of 4 mcg/mL for up to 3 h, but was not sufficient for 4 h. The Monte Carlo simulation method used in this study showed that 2-g and 3-g doses were not likely to achieve a MIC for more than 2 h, and only a 3-g bolus plus 1-g infusion was sufficient in staying above the MIC for 4 or more hours. [35]

**Table 4:** Key findings of pharmacokinetic studies without a comparator group

Study	Surgery type	Participants (***N***)	Patient characteristics	MIC	Measured tissue	Outcome	2-g dose sufficient?
van Kralingen et al. 2011 [33]	Bariatric surgery (gastric banding and gastric bypass)	20	2 g of cefazolin, mean BMI 51.0 kg/m^2^	1 mcg/mL	Interstitial/plasma	Unbound plasma cefazolin concentrations remained above MIC for 4 h	Yes
Chen et al. 2017 [32]	Bariatric surgery (gastric bypass and sleeve gastrectomy)	37	2 g cefazolin, BMI ≥ 35 kg/m^2^	1 mcg/mg	Interstitial/plasma and Adipose	Cefazolin concentration always exceeded MIC	Yes
Gregoire et al. 2018 [35]	Bariatric surgery (sleeve gastrectomy)	117	4 g of cefazolin, BMI > 40 kg/m^2^	4 mcg/mL and 2 mcg/mL	Interstitial/plasma	Simulated 2 g and 3 g regimens do not provide adequate coverage. 3 g bolus + 1 g infusion gives best results	No
Eley et al. 2020 [34]	Caesarean section	12	2 g of cefazolin, BMI ≥ 35 kg/m^2^	2 mcg/mL	Interstitial/plasma	MIC was not maintained in 2/12 patients. Simulations showed that changing dose to 3 g improves this	No

summarises key information relating to pharmacokinetic studies that did not contain multiple real experimental groups, including doses, MIC targets named, tissue type that was sampled and conclusions drawn

## Discussion

Cefazolin is the most widely used drug for SSI prophylaxis [11, 12]. Although it has a wide therapeutic window [36, 37], the use of unnecessarily high doses increases costs, increases selection pressure on mutations for resistance [38, 39] and could increase the risk of *C. difficile* infection [40]. Conversely, under-dosing increases the risk of infection and resistance. Current guidelines on prophylactic cefazolin dosing for patients with obesity do not give consistent recommendations. For instance, the British National Formulary [11] recommends 1 g given before surgery followed up with another 0.5–1 g after 2 h, without any adjustment for body weight. In contrast, the Australian Therapeutic Guidelines [12] recommend 2 g in adults, and 3-g dose “is reasonable” in patients weighing more than 120 kg, citing studies that draw mixed conclusions, including some that do not recommend a change in dosage [18, 23, 28]. Resources such as those published by UpToDate [41] mimic American Society of Health-System Pharmacists [10, 42] guidelines recommending 3-g cefazolin dose if the patient weighs more than 120 kg. This recommendation is based on few studies, with only one that directly investigated cefazolin dosing in patients with obesity. [31]

Although most studies included in this review assessed pharmacokinetic data, prevention of infection is the primary goal of the prophylactic dosing strategy, and outcome studies provide the real-world outcome data. Two outcome studies concluded that above 2-g dose is neither required nor efficacious [18, 19]. A third study [20] found non-statistically significantly higher SSI rates in the obese cohort compared to the non-obese cohort, who all received 2 g. Overall, outcome studies do not support prophylactic doses higher than 2 g cefazolin in surgical patients with obesity.

Drawing conclusions from pharmacokinetic studies is more complex than from outcome studies, as a greater number of variables are assessed in these studies. Forms of heterogeneity between studies, such as type of surgery, cefazolin doses given, participant characteristics, MIC targets adopted, sampling methods and the simulation approaches, result in increased complexity. Grouping studies by such categories may help discern whether any of these factors predicted a particular conclusion.

The most evident factor for potential variation between patients is the weight categories included. Studies that solely examined different doses between groups [16, 17, 23–26, 34, 35] had wide mean BMI ranges (38.9–49.7 kg/m^2^) and reported inconsistent findings. For instance, the study that included groups of patients from the heaviest weight ranges (BMI 40–49 kg/m^2^ as the lowest weight group and BMI 50–59 kg/m^2^ and > 60 kg/m^2^ groups) concluded that higher doses are needed for patients with obesity [31], but another that included patients with a BMI range of 38–79 kg/m^2^2, 33 concluded that 2 g is sufficient.

Another potential reason for varied findings is that different studies used different MIC values (1 to 32 mcg/mL). Two studies chose MIC targets > 8 mcg/mL (or > 8 mcg/g), with one concluding that 2 g of cefazolin is sufficient [28] and the other concluding that 2 g is not sufficient [31]. Studies at the other end of the spectrum which used a defined MIC value of 1 mcg/mL [32, 33] to cover staphylococcal species in gastric bypass surgeries found that 2 g of cefazolin was sufficient. Studies between these two extremes, which selected MIC targets of 2–8 mcg/mL (or 2–8 mcg/g in adipose tissue), had mixed conclusions with five concluding that 2 g is not sufficient [17, 27, 34, 35, 39] and six concluding that a 2-g dose is sufficient. [16, 23–26, 29]

Out of six pharmacokinetic studies which concluded that 2-g dose was insufficient in individuals with obesity, three used Monte Carlo simulation techniques [30, 34, 35]. Conversely, of the nine pharmacokinetic studies concluding that 2 g is sufficient, only one used Monte Carlo simulations [24]. As such, use of simulation techniques appears to be predictive of the conclusion that a 2-g dose is insufficient. As with all statistical or probabilistic-based conclusions, accurate Monte Carlo simulations have two prerequisites: (1) an unbiased sample and (2) a sufficient sample size [15]. If there is bias in the sample on which the simulations are based, this will be reflected and perhaps amplified in the simulated outcome. Also, if the sample size is insufficient, one has less power to draw conclusions on the biassed nature of the simulation. This caveat is paradoxical, as Monte Carlo simulations are used as a way of ‘expanding’ sample sizes to predict outcomes [15]. Of the four studies using simulated patients, three had an actual patient sample size of 15 or less [25, 30, 34]. This may represent a significant weakness in these study designs. A limitation of the Monte Carlo study which based its simulations on a larger sample (*N* = 117) [35] is that it included only high (4 g) doses of cefazolin given to real patients, perhaps representing a significant source of bias. In summary, although a higher proportion of simulation studies concluded that the dose of cefazolin should be increased in patients with obesity, these study designs carry inherent limitations.

The principles of antibiotic prophylaxis remain the same between different surgical procedures, and multiple guidelines recommend the same doses of cefazolin across many types of surgery [11, 12, 41]. However, several factors, such as wound type, invasive nature of the surgery (laparoscopic surgery vs open surgery) and type of tissue being operated on, can alter the risk of SSI [43]. Eight studies included in this review were conducted on caesarian delivery patients,^17,18,23,24,26–28,34^, seven on bariatric surgery patients [25, 29–33, 35] and three on patients undergoing mixed elective surgical procedures such as orthopaedic, gynaecological and trauma-related [16, 19, 20]. Three of the bariatric (laparoscopic) surgery studies [30, 31, 35] and three of the caesarian delivery studies [17, 27, 34] concluded that 2 g of cefazolin was insufficient, indicating that neither surgery type appears to predict an increased need for greater doses of cefazolin over the other. Hence, it can be concluded that type of surgery does not appear to warrant a need to alter the cefazolin dose.

A final important consideration in assessment of conclusions is quality of included studies. All three randomised control studies included [23, 24, 26] in this review concluded that there is no need for higher dose or re-dosing of cefazolin in patients with obesity. Cohort studies included in this review reported mixed findings: half of these studies concluded that 2 g of cefazolin was sufficient [16, 25, 28, 29] and half concluded the need for an increased dose in patients with obesity [17, 27, 30, 31]. Case series studies also reported an even split in their conclusions [32–35]. Finally, all case control studies (i.e. outcome studies)^18–20^ concluded that 2-g cefazolin dose is sufficient to provide adequate SSI prophylaxis in patients who are obese. However, there is no outcome-based RCT; findings of these outcome studies should be considered with caution.

There are a few important limitations of this review. Firstly, there was a high degree of heterogeneity between studies, such as in study design, sampling techniques, sampling tissue, MIC cutoff values and study populations. Such variation can make it difficult to directly compare studies. Secondly, outcome studies comparing the broad family of general surgery between normal BMI with obese and morbidly obese do not compare procedures for the same diagnosis and the same approach which cause a real bias. Thirdly, due to this heterogeneity, meta-analysis could not be conducted, leaving us with a mostly qualitative analysis. Fourthly, although outcome studies were assessed, only three such studies matching selection criteria were found, again potentially reducing the generalisability of this review to real-world clinical settings. Fifthly, as there were no large-scale registry studies included in this review, sample sizes were relatively small. Finally, the surgery duration for the majority of studies included in this systematic review was less than 4 h. Therefore, the findings of this review will need to be carefully applied to prolonged surgeries of more than 4-h duration.

## Conclusion

Despite consistent findings that cefazolin tissue concentrations are inversely correlated with BMI of the patient, [27, 29, 30, 32] the bulk of evidence supports the notion that there is no need for higher doses of cefazolin for SSI prophylaxis in patients with obesity for surgical procedures lasting up to 4 h. All outcome studies and nine out of fifteen pharmacokinetic studies included in this review (including all three RCTs) reported this conclusion. If studies relying on simulation techniques are discounted, only three out of fourteen recommend a dose increase. Factors such as type of tissue sampled and type of surgical procedure did not appear to influence the success of using a 2-g dose of cefazolin. Although large-scale outcome-based RCTs are needed in the area, current evidence does not support higher than 2-g prophylactic cefazolin doses in surgical patients with obesity.

## Supplementary Information

Below is the link to the electronic supplementary material.

Supplementary file1 (DOCX 49 KB)
