# Supplementary material for: Prophylactic Cefazolin Dosing in Obesity—a Systematic Review
Source: Obes Surg. 2022 Jul 9;32(9):3138–49. doi: 10.1007/s11695-022-06196-5 (PMC9392691; doi:10.1007/s11695-022-06196-5)
Supplement: Supplementary file 1 — Supplementary file1 (DOCX 49 KB) [file 11695_2022_6196_MOESM1_ESM.docx]

**Supplementary data**
**Search Terms**

The following terms were submitted to Scopus, MedLine, PubMed, and CINAHL databases on 13/04/21:
(TX Cefazolin OR TX Cephazolin OR TX Cefazoline) AND (TX Obese OR TX Obesity OR TX Morbidly Obese OR TX Overweight) AND (TX Surgery OR TX Surgical OR TX Elective OR TX Procedure) AND (TX Prophylaxis OR TX Prophylactic OR TX Dose)

**Appendix 1 – PRISMA Checklist**

| **Section and Topic** | **Item #** | **Checklist item** | **Location where item is reported** |
| --- | --- | --- | --- |
| **TITLE** | | |  |
| Title | 1 | Identify the report as a systematic review. | Title Page |
| **ABSTRACT** | | |  |
| Abstract | 2 | See the PRISMA 2020 for Abstracts checklist. | Abstract |
| **INTRODUCTION** | | |  |
| Rationale | 3 | Describe the rationale for the review in the context of existing knowledge. | Introduction |
| Objectives | 4 | Provide an explicit statement of the objective(s) or question(s) the review addresses. | Introduction |
| **METHODS** | | |  |
| Eligibility criteria | 5 | Specify the inclusion and exclusion criteria for the review and how studies were grouped for the syntheses. | Methods, results |
| Information sources | 6 | Specify all databases, registers, websites, organisations, reference lists and other sources searched or consulted to identify studies. Specify the date when each source was last searched or consulted. | Methods, Figure S1 |
| Search strategy | 7 | Present the full search strategies for all databases, registers, and websites, including any filters and limits used. | Figure S1 |
| Selection process | 8 | Specify the methods used to decide whether a study met the inclusion criteria of the review, including how many reviewers screened each record and each report retrieved, whether they worked independently, and if applicable, details of automation tools used in the process. | Methods |
| Data collection process | 9 | Specify the methods used to collect data from reports, including how many reviewers collected data from each report, whether they worked independently, any processes for obtaining or confirming data from study investigators, and if applicable, details of automation tools used in the process. | Methods |
| Data items | 10a | List and define all outcomes for which data were sought. Specify whether all results that were compatible with each outcome domain in each study were sought (e.g., for all measures, time points, analyses), and if not, the methods used to decide which results to collect. | Methods |
|  | 10b | List and define all other variables for which data were sought (e.g., participant and intervention characteristics, funding sources). Describe any assumptions made about any missing or unclear information. | Methods |
| Study risk of bias assessment | 11 | Specify the methods used to assess risk of bias in the included studies, including details of the tool(s) used, how many reviewers assessed each study and whether they worked independently, and if applicable, details of automation tools used in the process. | Methods |
| Effect measures | 12 | Specify for each outcome the effect measure(s) (e.g., risk ratio, mean difference) used in the synthesis or presentation of results. |  |
| Synthesis methods | 13a | Describe the processes used to decide which studies were eligible for each synthesis (e.g., tabulating the study intervention characteristics and comparing against the planned groups for each synthesis (item #5)). | Methods, Results |
|  | 13b | Describe any methods required to prepare the data for presentation or synthesis, such as handling of missing summary statistics, or data conversions. | Methods |
|  | 13c | Describe any methods used to tabulate or visually display results of individual studies and syntheses. | Methods |
|  | 13d | Describe any methods used to synthesize results and provide a rationale for the choice(s). If meta-analysis was performed, describe the model(s), method(s) to identify the presence and extent of statistical heterogeneity, and software package(s) used. | Methods |
|  | 13e | Describe any methods used to explore possible causes of heterogeneity among study results (e.g., subgroup analysis, meta-regression). | Methods |
|  | 13f | Describe any sensitivity analyses conducted to assess robustness of the synthesized results. |  |
| Reporting bias assessment | 14 | Describe any methods used to assess risk of bias due to missing results in a synthesis (arising from reporting biases). | Methods |
| Certainty assessment | 15 | Describe any methods used to assess certainty (or confidence) in the body of evidence for an outcome. |  |
| **RESULTS** | | |  |
| Study selection | 16a | Describe the results of the search and selection process, from the number of records identified in the search to the number of studies included in the review, ideally using a flow diagram. | Results |
|  | 16b | Cite studies that might appear to meet the inclusion criteria, but which were excluded, and explain why they were excluded. |  |
| Study characteristics | 17 | Cite each included study and present its characteristics. | Results |
| Risk of bias in studies | 18 | Present assessments of risk of bias for each included study. | Methods, Tables S2-S4 |
| Results of individual studies | 19 | For all outcomes, present, for each study: (a) summary statistics for each group (where appropriate) and (b) an effect estimates and its precision (e.g., confidence/credible interval), ideally using structured tables or plots. | Tables S5-S6 |
| Results of syntheses | 20a | For each synthesis, briefly summarise the characteristics and risk of bias among contributing studies. | Results |
|  | 20b | Present results of all statistical syntheses conducted. If meta-analysis was done, present for each the summary estimate and its precision (e.g., confidence/credible interval) and measures of statistical heterogeneity. If comparing groups, describe the direction of the effect. |  |
|  | 20c | Present results of all investigations of possible causes of heterogeneity among study results. | Discussion |
|  | 20d | Present results of all sensitivity analyses conducted to assess the robustness of the synthesized results. |  |
| Reporting biases | 21 | Present assessments of risk of bias due to missing results (arising from reporting biases) for each synthesis assessed. |  |
| Certainty of evidence | 22 | Present assessments of certainty (or confidence) in the body of evidence for each outcome assessed. |  |
| **DISCUSSION** | | |  |
| Discussion | 23a | Provide a general interpretation of the results in the context of other evidence. | Discussion |
|  | 23b | Discuss any limitations of the evidence included in the review. | Discussion |
|  | 23c | Discuss any limitations of the review processes used. | Discussion |
|  | 23d | Discuss implications of the results for practice, policy, and future research. | Discussion |
| **OTHER INFORMATION** | | |  |
| Registration and protocol | 24a | Provide registration information for the review, including register name and registration number, or state that the review was not registered. |  |
|  | 24b | Indicate where the review protocol can be accessed, or state that a protocol was not prepared. |  |
|  | 24c | Describe and explain any amendments to information provided at registration or in the protocol. |  |
| Support | 25 | Describe sources of financial or non-financial support for the review, and the role of the funders or sponsors in the review. |  |
| Competing interests | 26 | Declare any competing interests of review authors. | Title page |
| Availability of data, code and other materials | 27 | Report which of the following are publicly available and where they can be found: template data collection forms; data extracted from included studies; data used for all analyses; analytic code; any other materials used in the review. | Methods, Supplement materials |

*From:*  Page MJ, McKenzie JE, Bossuyt PM, Boutron I, Hoffmann TC, Mulrow CD, et al. The PRISMA 2020 statement: an updated guideline for reporting systematic reviews. BMJ 2021;372:n71. doi: 10.1136/bmj.n71

For more information, visit: <http://www.prisma-statement.org/>

**Appendix 2 – JBI tables: Quality Assessment for case control studies**

|  | Ahmadzia et al 2015 | Peppard et al 2016 | Hussain et al 2019 |
| --- | --- | --- | --- |
| 1. Were the groups comparable other than the presence of disease in cases or the absence of disease in controls? | y - aside from a mean BMI difference of 3, groups were comparable. (Table 1) | n - 3g group had higher BMI, rates of diabetes, immunosuppressive medication | y - aside from diabetes rate, weight was the only major difference between groups |
| 2. Were cases and controls matched appropriately? | unsure - no discussion of matching | y | y |
| 3. Were the same criteria used for identification of cases and controls? | y - only dosing was different | y - different doses | y - body mass |
| 4. Was exposure measured in a standard, valid and reliable way? | y - dose of cefazolin | y - dose of cefazolin | y - body mass |
| 5. Was exposure measured in the same way for cases and controls? | y - dose administered | y - dose administered | y - body mass |
| 6. Were confounding factors identified? | y - second final paragraph | n- no mention of other conditions or drugs, and Patients were included even if the timing of cefazolin fell outside the SCIP guideline of zero to 60 minutes before incision. | y - " (i) lacked follow-up within 90 days of surgery, (ii) had an unplanned non-infective post-operative intensive care unit admission, (iii) had a second operation during the same admission for causes other than infection, (iv) required perioperative blood transfusion, (v) were taking systemic immunosuppressive medication (corticosteroids, sirolimus, everolimus, cyclosporine, tacrolimus, azathioprine, mycophenolate, monoclonal antibodies or biologics, e.g. abatacept, etanercept) at admission and/or discharge, (vi) were receiving antibiotics immediately prior to admission or (vii) had missing requisite data" |
| 7. Were strategies to deal with confounding factors stated? | y - exclusion | n | y - exclusion |
| 8. Were outcomes assessed in a standard, valid and reliable way for cases and controls? | y - rates of SSI (defined by CDC criteria) in 30 days following surgery | y - SSI by documentation of medical record | y - rates of SSI as per hospital records over 90-day follow-up |
| 9. Was the exposure period of interest long enough to be meaningful? | y - as per guidelines | y - as per guidelines | y - as per guidelines |
| 10. Was appropriate statistical analysis used? | y - OR and significance testing | y - OR and significance testing | y - "Pearson’s χ2 test and Fisher’s exact test were used for categorical variables, and the Mann-Whitney U test was used for continuous variables to compare the baseline variables and primary outcome." |

**Green shaded cells indicate that a JBI criterion was met. Red indicates that a JBI criterion was not met. Yellow indicates that more information is needed.**

**Appendix 3 – JBI tables: Quality Assessment for RCTs**

|  | Maggio et al 2015 | Young et al 2015 | Stitely et al 2013 |
| --- | --- | --- | --- |
| 1. Was true randomisation used for assignment of participants to treatment groups? | y - "Permuted block randomization with varying block sizes was used to stratify randomization by patient BMI: 30–39.9 and greater than 40. The randomization list was managed and maintained by the pharmacy staff. All other investigators, study participants, operating room staff, and anesthesiologists were blinded to group allocation." | y - "Randomization was performed by the pharmacy using www.randomizer.org, using 1:1 simple randomization" | y - "Subjects were randomized using a simple scheme employing computer-generated random numbers, with the allocation concealed until randomization using sequentially numbered opaque envelopes" |
| 2. Was allocation to treatment groups concealed? | y | y - "All investigators and participants were blinded to the dose assignment" | n - "Subjects, medical personnel, investigators, and laboratory personnel were not blinded to allocation once randomization was complete" |
| 3. Were treatment groups similar at the baseline? | y - aside from rate of gestational diabetes, dose was the only difference (table 1) | y - seen in table 1 | y - seen in table 1 |
| 4. Were participants blind to the treatment assignment? | y | y - "All investigators and participants were blinded to the dose assignment" | n |
| 5. Were those delivering treatment blind to treatment assignment? | y - only pharmacy staff saw | y - "All investigators and participants were blinded to the dose assignment" | n |
| 6. Were outcomes assessors blind to treatment assignment? | y - only pharmacy staff saw | y - "All investigators and participants were blinded to the dose assignment" | n |
| 7. Were treatment groups treated identically other than the intervention of interest? | y - all assessors were blinded | y - all assessors were blinded | y - seen under "Plasma and Tissue Sample Collection and Analysis" section (although there was no blinding) |
| 8. Was follow up complete and if not, were differences in follow up between groups adequately described and analysed? | y - "There were similar follow-up rates in the 2-g and 3-g groups (29% compared with 35%, P=.63)" | y | y |
| 9. Were participants analysed in the groups to which they were randomised? | y | y | y |
| 10. Were outcomes measured in the same way for treatment groups? | y - tissue samples and opening and closure | y - outlined in section D of methods | y - seen under "Plasma and Tissue Sample Collection and Analysis" section |
| 11. Were outcomes measured in a reliable way? | y - cefazolin concentration | y - cefazolin concentration | y - cefazolin concentration |
| 12. Was appropriate statistical analysis used? | y - analysis of variance | y - outlined in section F of methods | y - "Statistical analysis was performed using JMP 7.0 (SAS Institute Inc., Cary, NC) and Excel 2007 statistics package add-in (Microsoft, Redmond, WA). Continuous data were analyzed using paired and unpaired t-tests as appropriate, and categorical data were analyzed by the Fisher exact test. In situations where data failed a test of normality (Kolmogorov–Smirnov test), a non-parametric (Mann–Whitney U) test was used." |
| 13. Was the trial design appropriate, and any deviations from the standard RCT design (individual randomization, parallel groups) accounted for in the conduct and analysis of the trial? | y | y | y |

**Green shaded cells indicate that a JBI criterion was met. Red indicates that a JBI criterion was not met. Yellow indicates that more information is needed.**

**Appendix 4 – JBI tables: Quality Assessment for cross-sectional studies**

|  | Anlicoara et al 2014 | Brill et al 2014 | Edmiston et al 2017 |
| --- | --- | --- | --- |
| 1. Were the criteria for inclusion in the sample clearly defined? | unsure - following the "criteria of the Brazilian Society of Bariatric and Metabolic Surgery" | y - "Morbidly obese patients (BMI >40 kg/m2) undergoing laparoscopic gastric bypass surgery and non-obese patients (BMI 20–30 kg/m2 at inclusion in the study) undergoing laparoscopic Toupet fundoplication surgery were considered for inclusion in the study" | n - mentions criteria, but doesn't define it |
| 2. Were the study subjects and the setting described in detail? | n - only the selection criteria | y - characteristics summarised in table 1 | y - outline in table 1 |
| 3. Was the exposure measured in a valid and reliable way? | y - BMI | y - BMI | y - BMI |
| 4. Were objective, standard criteria used for measurement of the condition? | y - BMI | y - BMI | y - BMI |
| 5. Were confounding factors identified? | y - "Patients with hypotension who required the use of vasoactive drugs during surgery and those with altered renal function (creatinine >1.5 mg/dl) were excluded from the study" | y - "Patients were excluded from the study if they were pregnant, breastfeeding, suffered from renal insufficiency, had a known allergy to cefazolin or had an ejection fraction <35%" | n - exclusions were based on failure to obtain samples |
| 6. Were strategies to deal with confounding factors stated? | y - exclusion | y - exclusion | n |
| 7. Were the outcomes measured in a valid and reliable way? | y - cefazolin concentration | y - cefazolin concentration | y - cefazolin concentration |
| 8. Was appropriate statistical analysis used? | n - mean and SD stated, but no significance testing | y - "Student's t-test was applied to test differences in demographic variables between the study groups. For cefazolin concentrations the non-parametric Mann–Whitney test was applied to test statistical differences between the groups" | y - "Descriptive statistical analysis, ANOVA, and chi-square tests were performed on selected data sets using the Minitab Statistical Program, release 13 (State College, Pa)" |

|  | Groff et al 2017 | Palma et al 2018 | Pevzner et al 2011 |
| --- | --- | --- | --- |
| 1. Were the criteria for inclusion in the sample clearly defined? | y - "Women who were 18 years of age or older, greater than 37 weeks' gestation, and scheduled to deliver via C/S before onset of labor or rupture of membranes were consented and included in this study" | y - "The inclusion criteria were: patients aged 18 years and older, with a BMI ≥ 40 kg/m2 or BMI ≥ 35 kg/m2 with comorbidities and submitted to open bariatric surgery (gastric bypass or sleeve gastrectomy)" | y - "Individuals scheduled for cesarean delivery at term (more than 37 completed weeks of gestation) under nonemergent circumstances were eligible for participation in study" |
| 2. Were the study subjects and the setting described in detail? | n - only BMI and gestational age were described | y - table 1 | y - table 1 |
| 3. Was the exposure measured in a valid and reliable way? | y - BMI | y - 2g or 3g dose prior to surgery | y - BMI |
| 4. Were objective, standard criteria used for measurement of the condition? | y - BMI | y - dose | y |
| 5. Were confounding factors identified? | y - "Women with multiple gestation pregnancy, allergy to penicillin or other cephalosporins, exposure to other antibiotics within 24 hours before the scheduled C/S, history of illicit substance use, diagnosis of polyhydramnios, fetal or chromosomal defects or major anomalies, signs of infection at the time of delivery, or diagnosis of any of the following disorders including maternal hypertensive disorders, maternal diabetes, renal insufficiency (serum creatinine > 1 mg/dL), and chronic infections—including hepatitis B, hepatitis C, or HIV—were excluded from this study." | y - "renal and/or hepatic dysfunction, antimicrobial treatment and/or allergy to cephalosporins" | y - "known cephalosporin allergy, exposure to antibiotics within 7 days before the cesarean delivery, need for emergent delivery, active labor, multiple gestations, suspected chorioamnionitis, and medical complications that could theoretically result in microvascular disease, which could potentially affect the pharmacokinetics and pharmacodynamics of prophylactic antibiotics" |
| 6. Were strategies to deal with confounding factors stated? | y - exclusion | y - exclusion | y - exclusion |
| 7. Were the outcomes measured in a valid and reliable way? | y - cefazolin concentration | y - "A POPPK model with variable plasma and subcutaneous tissue protein binding was developed to simultaneously describe plasma and tissue data sets" | y - cefazolin concentration in adipose tissue |
| 8. Was appropriate statistical analysis used? | y - "MB and UCB concentrations were plotted against maternal BMI for both total and free concentrations of cefazolin to assess for a difference between the 2 groups of women within the study. Linear regression analysis was performed to determine the relationship between BMI and cefazolin concentration. Free cefazolin concentrations in MB and UCB were compared against the MIC required to inhibit 90% of growth for several pathogens" | y - presence of significance testing with POPPK modeling | y - Statistical analyses were performed using JMP 8.0 statistical software, and all tests were conducted at the 0.05 significance level. The Dunnett test was used to test differences in means from the three BMI categories with the BMI less than 30 group as the control mean. Normality of continuous data were assessed by the Shapiro-Wilk test. Continuous data that were not normally distributed were compared with the Kruskal-Wallis rank-sum test. Categorical variables were evaluated using the Fisher exact test. |

|  | Ho et al 2012 | Swank et al 2015 | Chen et al 2017 |
| --- | --- | --- | --- |
| 1. Were the criteria for inclusion in the sample clearly defined? | n - just "enrolled according to institutional informed consent guidelines" | unsure - "identical to in Pevzner 2011", but not defined within paper | y - "The study recruited 37 patients undergoing Roux-en-Y gastric bypass (RYGB) or laparoscopic sleeve gastrectomy (LSG), aged 18–60 years old, with a body mass index (BMI) ≥ 35 kg/m2" |
| 2. Were the study subjects and the setting described in detail? | y - table 1 | y - table 1 | y - "Thirty-seven patients completed the study, 11 men (30 %) and 26 women (70 %). The mean age was 45 + 13 years old, mean weight was 127 + 29 kg, and mean BMI was 46 + 8 kg/m2. The majority of the patients were Caucasian (92 %) while only three patients were African-American (8 %)" |
| 3. Was the exposure measured in a valid and reliable way? | y - BMI and dose | y - defined by BMI and dose of cefazolin | y - 2g cefazolin |
| 4. Were objective, standard criteria used for measurement of the condition? | y - BMI and dose | y | n - only one group |
| 5. Were confounding factors identified? | y - "Patients with allergy to penicillin or cephalosporins, current pregnancy or lactation, chronic kidney disease, or chronic hepatic insufficiency were excluded" | y - "any chronic medical comorbidity that potentially could affect tissue perfusion, thereby influencing pharmacokinetics; these included chronic hypertension, pregestational diabetes mellitus, and collagen vascular diseases including systemic lupus erythematosus. Other exclusion criteria included patient allergy to cephalosporins, exposure to antibiotics within the 7 days preceding cesarean delivery, multiple gestations, need for emergent delivery, or suspected preexisting infection." | y - "Patients who were pregnant, had moderate renal impairment (serum creatinine > 1.5 mg/dl), or were allergic to penicillin were excluded" |
| 6. Were strategies to deal with confounding factors stated? | y - exclusion | y - exclusion | y - exclusion |
| 7. Were the outcomes measured in a valid and reliable way? | y - Serum cefazolin concentrations were measured 5, 30, 120, and 360 min after initiation of the dose. An MIC of 8mcg/mL was used. | y - Serum cefazolin concentrations | y - Cefazolin concentrations, taken from serum, subcutaneous adipose tissue, and deep peri-gastric adipose tissue specimens at incision and before skin closure. An MIC of 1mg/L was used (S. aureus) |
| 8. Was appropriate statistical analysis used? | y - "Patient group characteristics were analyzed using measures of central tendency and the Kruskal-Wallis one-way analysis of variance. The elimination rate constant (ke) was calculated using a linear regression of the terminal portion of the concentration time profile, and the half-life was determined by 0.693/ke. All calculations were performed using STATA version 11.0 (Stata Corp., College Station, TX). The fT>MIC was determined using a protein binding value of 85%. The fT>MIC of 8 mcg/mL was determined for each patient, as was the protective duration of each regimen using the pharmacodynamic target of fT>MIC of 70%" | unsure - no significance testing, but IQR presented | n - no significance testing |

|  | Eley et al 2020 | Gregoire et al 2018 | van Kralingen et al 2011 |
| --- | --- | --- | --- |
| 1. Were the criteria for inclusion in the sample clearly defined? | y - "Women were included if they had a BMI >35 kg·m−2 at delivery, age ≥18 years, were booked for an elective CD at a gestation of ≥37 weeks, and commencing under neuraxial anesthesia" | y - "Morbidly obese patients aged 18–70 years, with a body mass index (BMI) ≥40 and ≤65 kg/m2 and undergoing sleeve gastrectomy were considered for inclusion in the study" | y - "age between 18 and 60 years, American Society of Anaesthesiologists (ASA) physical status classification II or III, a BMI >35 kg m−2 at inclusion together with an indication for weight-reducing surgery and a normal renal and hepatic function as assessed by routine laboratory testing" |
| 2. Were the study subjects and the setting described in detail? | y - table 1 | y - table 1 | y - table 1 |
| 3. Was the exposure measured in a valid and reliable way? | y - 2g cefazolin | unsure - 4g dose is well above guidelines | y - 2g cefazolin |
| 4. Were objective, standard criteria used for measurement of the condition? | n - only one real human group | n - only one real human group | n - only one real human group |
| 5. Were confounding factors identified? | y - "elevated serum creatinine (>70 µmol·L−1),23 allergy to cefazolin, or had received cefazolin in the preceding 72 hours" | y - "pregnancy, ongoing treatment by cefazolin, moderate or severe renal impairment defined by a Modification of Diet in Renal Disease (MDRD) estimated glomerular filtration rate (eGFR) ≤60 mL/min or known β-lactam allergy" | y - "pregnancy, treatment with antibiotics and known allergy for cefazolin" |
| 6. Were strategies to deal with confounding factors stated? | y - exclusion | y - exclusion | y - exclusion |
| 7. Were the outcomes measured in a valid and reliable way? | y - cefazolin concentration and standardised modeling | y - cefazolin concentration and standardised modeling | y - Blood samples were collected up to 4 h post-dosing to determine total and unbound plasma cefazolin concentrations. An MIC of 1mg/L was used. |
| 8. Was appropriate statistical analysis used? | y - Monte Carlo analysis | y - Monte Carlo analysis | y - covered in methods section just before results |

**Green shaded cells indicate that a JBI criterion was met. Red indicates that a JBI criterion was not met. Yellow indicates that more information is needed.**

**Appendix 5 – Study Details – Outcome Studies**

| **Authors** | **Year** | **Study design** | **Study Population** | **Intervention and comparator** | **Diagnostic criteria** | **Outcome** | **Conclusion** |
| --- | --- | --- | --- | --- | --- | --- | --- |
| Ahmadzia et al | 2015 | Retrospective cohort (two hospitals) | Morbidly obese pregnant women undergoing caesarean delivery (median BMI=51.3) | 2g compared with 3g of cefazolin preoperatively | Presence or absence of SSI post-surgery using CDC criteria | Cohort 1- 13.1% of patients were diagnosed with SSI, regardless of 2g or 3g dosage. Cohort 2- 14.3% of patients with 2g dosage and 12.4% with 3g dosage had SSI | No difference between 2g and 3g dosages |
| Peppard et al | 2016 | Retrospective review | Patients ≥100 kg who were prescribed cefazolin as surgical prophylaxis at an academic medical centre | Group I: 152 patients treated with 2g of cefazolin; Group II: 284 patients treated with 3g of cefazolin | Rates of SSI up to 99 days post-surgery. The SSIs were identified by documentation of SSI in the medical record or findings consistent with the standard Centers for Disease Control and Prevention definition | “SSI rates were 7.2% and 7.4% (odds ratio [OR] 0.98, p = 0.95), for respective 2g and 3g groups. There was no difference in SSI rates (OR 0.87, 95% confidence interval 0.36-2.06, p = 0.77)” | There was no significant difference in SSI rates between 2g and 3g doses |
| Hussain et al | 2019 | Retrospective case control study | Elective surgery patients, either obese or non-obese | Group I: non-obese patients (weight <120kg); Group II: obese patients (weight>120kg), all treated with 2g of cefazolin | Presence or absence of SSI post-surgery | “While not statistically significant, the prevalence of SSI in the group with obesity was higher than in the non-obese group (8.6% vs 4.6%; p = 0.25) and in patients weighing >/= 120 kg (n = 102) compared to those weighing < 120 kg (n = 202) (9.8% vs 5.0%; p = 0.17)” | Despite a non-significant trend showing a difference between groups, the null hypothesis was not rejected |

**Appendix 6 – Study Details – Pharmacokinetic Studies**

| Anlicoara et al | 2014 | Cross-sectional cohort study | Patients with obesity undergoing bariatric surgery, all given 2g cefazolin initially followed by 1g continuous infusion | Patients with a BMI <40 kg/m^2^ were compared with patients with a BMI >40 kg/m^2^ | Cefazolin concentration in adipose tissue samples, analysed by reverse phase high-pressure liquid chromatography. An MIC of 4mcg/mL was used | “Patients with BMI <40 kg/m^2^ had higher initial and final sample concentrations of cefazolin than patients with BMI ≥40 kg/m^2^. There was no surgical site infection (SSI) in any of the patients, and concentrations remained above MIC in all cases” | An inverse correlation between BMI and concentration of cefazolin in adipose tissue was observed. 2g bolus + 1g continuous infusion was sufficient in all patients |
| --- | --- | --- | --- | --- | --- | --- | --- |
| Brill et al | 2014 | Cross-sectional cohort study | Morbidly obese patients (BMI >40 kg/m^2^) undergoing laparoscopic gastric bypass surgery and non-obese patients (BMI 20–30 kg/m2 at inclusion in the study) undergoing laparoscopic Toupet fundoplication surgery | Patients with a BMI >40 kg/m^2^ were compared with patients with a BMI 20-30 kg/m2 | Bound and unbound cefazolin concentrations, taken before surgery and 5, 10, 30, 60, 120 and 240 min after the cefazolin iv dose. Monte Carlo simulations were used to compare to MIC of 2 and 4mg/L | “For unbound cefazolin, cefazolin AUC was significantly lower in obese patients than non-obese. AUC for unbound plasma cefazolin did not differ significantly between the patient populations. Monte Carlo simulations showed that the probability of attaining MIC values >4mg/L after 240 minutes differed significantly between groups” | Unbound cefazolin concentrations were higher in the non-obese group |
| Chen et al | 2017 | Cross-sectional cohort study | Patients undergoing Roux-en-Y gastric bypass (RYGB) or laparoscopic sleeve gastrectomy (LSG), aged 18–60 years old, with a body mass index (BMI) ≥ 35 kg/m^2^ | All patients were given 2g of cefazolin. There was no apparent comparator group | Cefazolin concentrations, taken from serum, subcutaneous adipose tissue, and deep peri-gastric adipose tissue specimens at incision and before skin closure. An MIC of 1mg/L was used (S. aureus) | “Penetration of cefazolin into adipose tissue was only 6–8 % of simultaneous serum levels. However, cefazolin tissue concentrations in all adipose tissue specimens met an MIC of 1mg/L” | 2g dose of cefazolin was adequate for coverage of methicillin-sensitive *S. aureus* |
| Edmiston et al | 2004 | Cross-sectional cohort study | Patients with morbid obesity undergoing gastric bypass surgery | Patients were divided into groups based on BMI. Group I: BMI=40-49 kg/m^2^; Group II: BMI=BMI=50-59 kg/m^2^; Group III: BMI>60 kg/m^2^). All patients were given 2g of cefazolin followed by a second 2g dose at 3 hours | Multiple timed serum (baseline; incision, 15, 30, 60 minutes; prior to second prophylactic dose; and closure) and tissue (skin, subcutaneous fat) specimens were collected, and cefazolin concentration analysed by microbiological assay. An MIC of 32mcg/mL was used | “Serum antimicrobial concentrations exceeded resistance breakpoint (32 mcg/mL) in 73%, 68%, and 52% of BMI groups I, II, and III, respectively. There was a significant decrease in cefazolin concentrations in groups II and III compared to I” | Current dosing strategies may not be adequate in the morbidly obese |
| Eley et al | 2020 | Cross-sectional cohort study | Caesarean delivery patients with a BMI>35 who were given prophylactic cefazolin | All patients were given 2g of cefazolin. There was no apparent comparator group | Total and unbound cefazolin concentrations in plasma and interstitial fluid were measured. An MIC of 2mg/L was used. Population pharmacokinetic modelling and Monte Carlo dosing simulations were performed | “In two patients (2/12), MIC was not maintained without redosing 2g cefazolin. Simulations demonstrated that FTA >95% was achieved in patients weighing 90-150kg by an initial 2 g dose with redosing of 2 g at 2 hours. FTA was improved to >99% when an initial 3 g dose was repeated at 2 hours” | Guidelines may need to be reassessed to include redosing in obese patients |
| Gregoire et al | 2018 | Cross-sectional cohort study | Morbidly obese patients (BMI>40) undergoing sleeve gastrectomy and receiving cefazolin | All patients were given 4g of cefazolin. Modelling and Monte Carlo simulations were used to compare this with probability of remaining above MIC for 2g, 3g and 3g+1g continuous infusion | Serum concentrations of cefazolin were measured, and modelling was carried out. MIC values of 2mg/L and 4mg/L were used | 4g appears sufficient for normal MIC values and surgery times, though 3g +1g continuous infusion is the only dosage to remain above an MIC of 4mg/L 90% of the time after 4 hours | 2g and 3g regimens do not provide adequate coverage. 4g is sufficient, 3g +1g CI gives best results |
| Groff et al | 2017 | Cross-sectional cohort study | Patients undergoing caesarean delivery who received prophylactic cefazolin | Patients were split into obese (>30kg/m^2^) and non-obese (<25kg/m^2^) categories and given 2g of cefazolin | Maternal and fetal plasma concentrations of cefazolin were measured. These were compared against MIC values of 1mg/L, 2mg/L, and 18mg/L (for Group B Streptococcus, *S. aureus*, and *E. coli*) | “Cefazolin concentrations were slightly lower in the non-obese group (except in umbilical samples, where they were higher), but both groups maintained sufficient concentrations to achieve MIC in maternal blood” | 2g is sufficient in both obese and non-obese groups |
| van Kralingen et al | 2011 | Cross-sectional cohort study | Morbidly obese patients undergoing laparoscopic gastric banding or gastric bypass surgery | All patients were given 2g of cefazolin. There was no apparent comparator group | Blood samples were collected up to 4 hours post-dosing to determine total and unbound plasma cefazolin concentrations. An MIC of 1mg/L was used | In all patients with bodyweights up to 260kg, unbound plasma cefazolin concentrations remained above MIC until 4 hours after the intravenous administration of a 2g cefazolin dose | Redosing is not needed |
| Maggio et al | 2015 | Randomised control trial | Women undergoing caesarean delivery with BMI>30 kg/m^2^ | Patients either received 2g or 3g of cefazolin prior to surgery | Adipose tissue samples taken from before opening and after closure were taken. MIC values of 2mcg/g and 8mcg/g were used | Patients given 2g dose were above MIC for of 8mcg/g 61% of the time, while for 3g it was 72%. All patients were above MIC of 2mcg/g | 3 g of cefazolin did not significantly increase adipose tissue concentration |
| Palma et al | 2018 | Cross-sectional cohort study | Women with BMI >35 kg/m^2^ submitted for open bariatric surgery | Patients either received 2g or 3g of cefazolin prior to surgery | Probes were used to collect tissue samples and measure cefazolin concentration. POPPK modelling and Monte Carlo simulations were used. MIC values of 1mg/L and 2mg/L were used | 2g dose is sufficient for bacteria <1mg/L. For species requiring >2mg/L, 2g is sufficient for up to 4h, while 3g gives better coverage after this | Up to 4h of surgery, 2g is gives sufficient coverage. 3g may be needed for longer surgeries |
| Pevzner et al | 2011 | Cross-sectional cohort study | Patients undergoing caesarean delivery and receiving cefazolin | Group I: non-obese (BMI<30); Group II: obese (BMI=30-40); Group III: morbidly obese (BMI>40). All were given 2g of cefazolin | Antibiotic concentrations from adipose samples, collected after skin incision and before skin closure, along with myometrial and serum samples, were analysed with microbiological agar diffusion assay. MIC values of 1mcg/g and 4mcg/g were used | “Cefazolin concentrations within adipose tissue obtained at skin incision were inversely proportional to maternal BMI (r=-0.67, P<0.001). All patients attained levels above an MIC of 1mcg/g, 20% and 30-44% of patients in respective obese and very obese categories were not above an MIC of 4mcg/g” | Current dosing strategies may not be adequate in the morbidly obese |
| Young et al | 2015 | Randomised control trial | Obese women undergoing caesarean delivery | Patients were either given 2g or 3g of cefazolin prior to surgery | Serial maternal plasma samples were obtained at specific time points up to 8 hours after drug administration. Umbilical cord blood was obtained after placental delivery. Maternal adipose samples were obtained prior to fascial entry, after closure of the hysterotomy, and after fascial closure. MIC values of 1mg/g and 4mg/g were used | “For every 1 kg/m^2^ increase in body mass index at time of caesarean delivery, there was an associated 13.77mcg/mL lower plasma concentration of cefazolin across all time points. However, cefazolin concentrations adipose were consistently above MIC for both gram-positive and gram-negative bacteria with both the 2g and 3g doses” | Both doses are adequate, despite differences in concentration |
| Ho et al | 2012 | Cross-sectional cohort study | Obese or morbidly obese patients undergoing elective surgical procedures who received prophylactic cefazolin | Group I: 2g cefazolin IV push; Group II: 2g cefazolin 30 min infusion; Group III: severe obesity with 2g IV infusion; Group IV: severe obesity given 3g infusion | Serum cefazolin concentrations were measured 5, 30, 120, and 360 min after initiation of the dose. An MIC of 8mcg/mL was used | “The mean cefazolin concentrations after 30 min were similar in all groups. Half-life was unaffected by administration methods. The protective duration was 5.1 h, 4.8 h, 5.8 h, and 6.8 h for respective groups” | 2g doses appear sufficient in all weight categories. Method of administration does not affect concentration |
| Stitely et al | 2013 | Randomised control trial | Obese patients (first trimester BMI>35) undergoing caesarean delivery and receiving cefazolin | Patients either received 2g or 4g of cefazolin prior to surgery | Blood and subcutaneous tissues were collected at the times of incision and closure. Myometrial biopsies were collected at uterine closure. An MIC of 4mcg/g was used | Tissue concentrations were significantly higher in the 4g dosage group, but all patients in both groups remained above MIC. There were no SSIs in either group | Dosage makes a difference to PK parameters, but we cannot draw conclusions on protective threshold, as all patients in small sample remained above set MIC |
| Swank et al | 2015 | Cross-sectional cohort study compared with historic controls (Pevzner et al) | Obese patients undergoing caesarean delivery | Current cohort received 3g of cefazolin. Historic cohort received 2g | Adipose samples were collected at both skin incision and closure. An MIC of 8mcg/mL was used | “Higher weight meant lower cefazolin concentrations. Subjects with a BMI of 30-40 kg/m^2^ had a median concentration of 6.5 mcg/g vs 22.4 mcg/g after respective 2g and 3g doses. Subjects with a BMI of >40 kg/m^2^ had a median concentration of 4.7 mcg/g vs 9.6 mcg/g after respective 2g and 3g doses. With 2 g of cefazolin, only 20% of the cohort with a BMI of 30-40 kg/m2 and none of the cohort with a BMI of >40 kg/m^2^ reached an MIC of ≥8 mcg/mL. With 3-g, all women with a BMI of 30-40 kg/m^2^ reached target MIC values; 71% of the women with a BMI of >40 kg/m^2^ attained this cut-off" | 3g doses should be used in obese women. 2g is sufficient for normal and overweight |
